# Reflections on the Optimal Use of eDiaries for Data Collection in Vaccine Clinical Trials

**DOI:** 10.2196/66971

**Published:** 2025-09-19

**Authors:** Ujwani Nukala, Sylvia Cho, Aneesha Suresh Sahu, Jessica Zhou, Whitney R Steele, Barbee I Whitaker, Hussein Ezzeldin

**Affiliations:** 1 Office of Biostatistics and Pharmacovigilance, Center for Biologics Evaluation and Research, Food and Drug Administration, Building 71, 10903 New Hampshire Avenue, Silver Spring, MD, 20993, United States, 1 2402052212

**Keywords:** digital health technologies, patient-reported outcomes, electronic diaries, paper diaries, data collection, vaccine safety and efficacy, eDiaries, vaccine, vaccination, clinical trials, digital diaries

## Abstract

Recent years have witnessed a transformative shift in the way patient-reported outcomes are captured. The increasing adoption of digital health technologies offers exciting possibilities for more efficient, engaging, and insightful data collection electronically. Regulators recommend that the source data captured electronically should adhere to Attributable, Legible, Contemporaneous, Original, and Accurate principles to ensure data quality and integrity and be compliant with regulatory requirements. Traditionally, paper diaries are used to collect safety data in clinical trials; however, electronic forms of these paper diaries represent a paramount resource that could improve data quality, reduce costs, and limit the burden on clinical staff and trial participants. Electronic diaries (eDiaries) offer significant advantages, but specific measures must be taken to ensure their optimal use. In this paper, we provide our reflections on key measures, such as programming the eDiary platform, training the trial staff and participants, and real-time monitoring of participant compliance, to leverage eDiaries for optimal data collection. By implementing the measures discussed in this paper, eDiaries can offer significant advantages for both trial participants and clinical investigators by ensuring the quality and integrity of the data collected.

## Introduction

Patient-reported outcomes (PROs) are defined as “any report of the status of a patient’s health condition that comes directly from the patient, without interpretation of the patient’s response by a clinician or anyone else” [1]. PROs provide a direct, patient-centered perspective on the treatment effects and their quality of life that is not fully captured by traditional clinical measures alone, enabling clinicians to tailor an individualized treatment plan and facilitating shared decision-making with the patient. The use of PROs in clinical trials to inform decision-making, cost-effectiveness analysis, clinical guidelines, and health policy has shown a clear increase over time [2]. PROs offer a valuable tool for safety data collection in clinical trials, complementing traditional clinical trial data and providing a comprehensive picture of the therapeutic’s safety profile. Traditionally, PROs are collected through paper diaries where patients fill out a set of questions on a paper form either at the clinic or at home. The main problem with paper diaries, among others, is the quality and accuracy of the data collected because of patients’ noncompliance and recall bias [3]. It is known that participants may fill out the paper diaries for multiple days right before the visit while waiting in the parking lot, which is called the “parking lot effect” [4]. Using online platforms to present questions electronically allows easy data collection and real-time monitoring of compliance.

**Figure 1.**
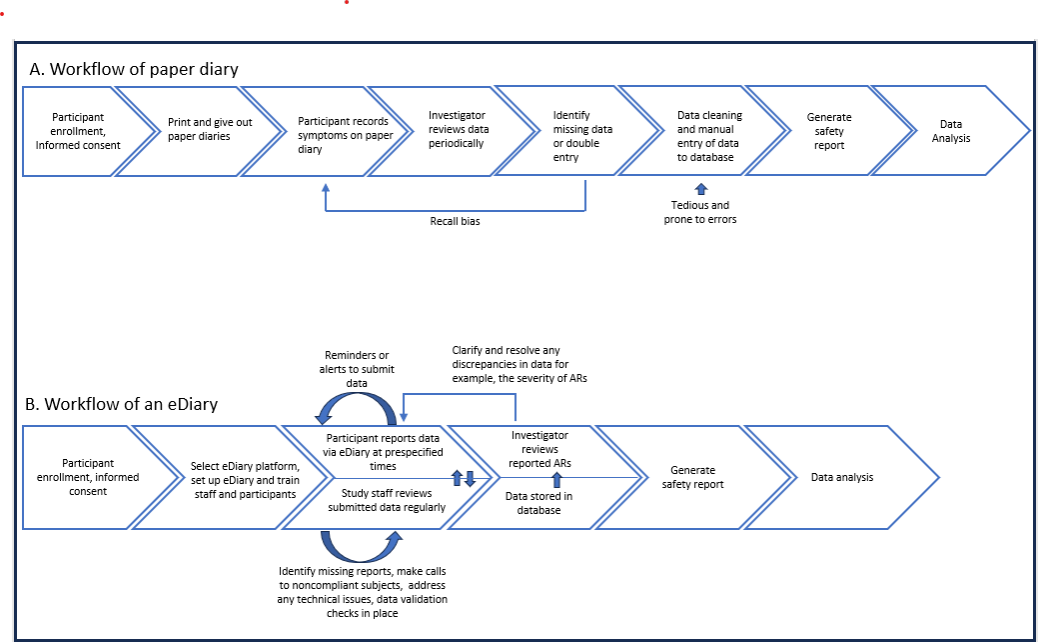
Workflows of (A) a traditional paper diary and (B) an eDiary for safety data collection in clinical trials. AR: adverse reaction; eDiary: electronic diary.

One of the most promising methods to collect electronic patient-reported outcomes (ePROs) is the use of digital health technologies such as electronic diaries (eDiaries). The term eDiary, or eDiaries, in our paper refers to the electronic form of the diary participants use, which has been increasingly used in clinical trials in general, and vaccine trials, in particular. Diaries, originally in paper form, are used to collect local and systemic solicited (predefined) adverse reactions (ARs) [5], and unsolicited (any additional other than the predefined) ARs post vaccination. Solicited and unsolicited ARs are standard primary safety endpoints in vaccine trials. In addition, the timing, or frequency, for collecting solicited and unsolicited ARs is predefined in the study protocols. In a few cases, eDiaries might be used to collect additional safety or efficacy measures such as patient-reported outcomes, eg, global impression severity or change; however, the collection of additional endpoints, or measures (other than the solicited and unsolicited ARs), is case dependent and is not standard across vaccine trials. The scope of this paper focuses on the most common use of eDiaries, which is the collection of solicited and unsolicited ARs. The paper and eDiary workflows are depicted in Figure 1, illustrating the benefits of an electronic data collection platform workflow compared to that of traditional paper diaries for the collection of safety data.

The US Food and Drug Administration’s (FDA) acceptance of data from clinical trials for decision-making purposes depends on the quality and integrity of the data [6]. As per title 21 Code of Federal Regulations Part 11, the FDA recommends that data should meet certain fundamental elements of quality whether collected electronically or on paper [7]. PROs data should adhere to Attributable, Legible, Contemporaneous, Original, and Accurate (ALCOA) standards and Complete, Consistent, Enduring, and Available (ALCOA+) criteria [8]. eDiaries can help ensure that the data collected in clinical trials meets these ALCOA principles, for example:

1. Attributable: It should be clear who documented the data. eDiaries can track who entered the data (participant or the clinical staff) and any modifications made, ensuring the data are attributable to a specific individual.
2. Legible: Data should be readable and understandable. Data collected using eDiaries are stored electronically, ensuring they are clear and easy to read.
3. Contemporaneous: Since eDiaries can be accessed on any general-purpose computing platform, such as a smartphone, computer, or tablet, participants can contemporaneously record the AR. Real-time reporting minimizes the need to recall symptoms later, which may lead to recall bias and incomplete and inaccurate data entry.
4. Original: eDiary can capture the original data, including any updates or corrections, providing a complete record of the data’s history.
5. Accurate: eDiaries can be programmed to include specific fields and prompts, guiding the participants to provide clear and specific information on each AR, including details such as severity and duration, to help minimize errors and ensure data accuracy.
6. Complete: By enforcing data validation rules via error messages (by setting specific criteria), implausible (defining acceptable ranges for numerical values or specific period), incomplete (setting mandatory fields before submission), missing data can be minimized to improve data quality and reliability [9].
7. Consistent: With built-in reminder systems in eDiaries, consistent data entry can be achieved and can be time-stamped. Use of eDiaries can trigger additional timely actions based on the identified case. These actions, usually outlined in the trial protocol, may include phone calls, site visits, or further assessments.
8. Enduring: Electronic storage of data collected using eDiaries is less susceptible to damage and is durable compared to paper diaries.
9. Available: Data stored electronically are readily available for analysis and auditing.

In addition, participants can conveniently access eDiaries anytime, anywhere, and on any general-purpose computing platform, providing the flexibility of remote data capturing and enabling recruitment of patients from diverse locations [10]. eDiaries can be programmed to send participants automatic reminders to report ARs within a specific reporting time window and can also be designed to have user-friendly and engaging interfaces, thus increasing the likelihood of all or most relevant events being captured, improving participant engagement, timely data reporting, and retention [5]. Finally, remote data capturing and cloud storage simplify the data handling process, increasing efficiency and reducing cost. The cost of implementation of a diary depends on the use case (eg, number of participants, length of trial, number of sites, support staff required, etc). As technology becomes more prevalent and more vendors enter the market, the cost, compared to paper diary, should be comparable, if not less than conventional PROs. As data collection processes typically associated with eDiaries are automated, eDiaries have the potential to lower the burden on trial staff. Reduced paperwork and streamlined data management can mitigate costs associated with data collection [11].

Despite the advantages, using eDiaries for data collection poses some challenges, including (1) participant engagement, (2) data quality and accuracy, (3) technology access, and (4) data privacy and security.

First, encouraging participants to be consistent with data reporting can be challenging because of language barriers, technology literacy, age-related factors such as cognitive ability or eyesight, or the length of the trial.

Second, as the data reported in eDiaries are self-reported, potential biases and inaccuracies may be introduced due to overreporting or underreporting of the symptoms, negatively impacting data quality and accuracy.

Third, having access to appropriate technology and internet connectivity can be challenging, particularly for participants from rural regions or low-income countries without access to general-purpose computing platforms [10].

Fourth, rigorous measures may be required to ensure data privacy and confidentiality.

Vaccines are generally administered to healthy populations; hence, there is a comparatively low tolerance for risk associated with vaccines relative to other pharmacological interventions. Any safety concerns, even if rare, require thorough investigation and mitigation strategies. Therefore, the collection of high-quality safety data in vaccine clinical trials is critical. eDiaries provide a structured and efficient method for capturing data and significantly improve the accuracy and reliability of the data collected. However, to fully realize the benefits of eDiaries, it is essential to address several key considerations that can harness the power of eDiaries. Optimal use of eDiaries, characterized by data quality and accuracy, patient engagement, and efficient data collection processes, can be achieved through the implementation of the measures discussed below (Table 1). These measures are proposed based on the expert opinion of the authors, from the experience gained reviewing several clinical trials, specifically vaccine clinical trials, where eDiaries were used to collect safety data, including solicited and unsolicited adverse events postvaccination.

**Table 1.** Strategies for optimizing eDiary^a^ use for data collection in clinical trials.

Elements	Proposed considerations
eDiary platform (user acceptance)	User-friendly (eg, intuitive login screen). User compatibility (eg, multiple font sizes and language options). Offline functionality. Accessible technical support. Compatibility with various general-purpose computing platforms.
Participant and trial staff training (human factors)	Educate participants on the importance of reporting ARs^b^ completely and accurately. Train participants to use eDiary (eg, hands-on tutorial, FAQs)^c^ Train trial staff on setting up participant accounts. Train trial staff to monitor participant compliance daily and follow up with noncompliant participants immediately. Train trial staff on how to troubleshoot any technical issues in the eDiary app.
Adherence and compliance (reminders, alerts [participant], and notifications [study personnel])	Reminders or alerts sent to participants to complete the eDiary. Notifications to trial staff with a list of noncompliant participants. Notifications to trial staff when a symptom of higher severity (grade 3 or more) is reported.
Data quality	Monitor data for its quality (eg, incomplete, implausible data). Ensure ALCOA^d^ standards [8]. Monitor daily compliance for any missing data.
Daily questionnaire	Comprehensive list of ARs to improve participant engagement. Clear and plain language to make participants comfortable using the eDiary. Accommodations for older participants, such as a bigger font size and additional training.
Reporting window	Select to balance recall bias and burden on participants. May be tailored depending on the trial protocol, its objective, and the trial population.
Date and time stamps (traceability)	Each eDiary should be labeled and date-stamped with its corresponding trial day (ie, day from last vaccination) along with the date and time stamps. Date and time stamp of when the eDiary was started should be recorded. Date and time stamp when the eDiary was completed or submitted should be recorded.
Data confidentiality and security	Have robust data security measures in place. Prevent unauthorized access to data.
Patient engagement	Provide regular feedback and acknowledge the participant’s contribution. Address any concerns participants may have in a timely manner. Collect feedback and improve data collection processes if necessary.

a eDiary: electronic diary.

b AR: adverse reaction.

c FAQ: frequently asked question.

d ALCOA: Attributable, Legible, Contemporaneous, Original, and Accurate.

## Reflections on the Optimal Usage of eDiaries in Vaccine Clinical Trials

### eDiary Platform

The eDiary platform should be clear, uncomplicated, and easy to navigate for participants with varying technical capabilities [12,13]. The username and password setup process should be straightforward and easy to understand. Screen reader compatibility and adjustable font size for visually impaired participants are some of the accessibility features to be considered [14]. To accommodate a broader participant pool, the interface should be offered in multiple languages. Offline functionality of the eDiary platform may be necessary to allow the participants to record ARs even when they do not have access to the internet, with automatic syncing to the server when a connection becomes available. Providing clear and accessible technical support details with the eDiary app would be beneficial in case of data reporting difficulties. Using an eDiary platform that is compatible with various general-purpose computing platforms is crucial [15].

### Training and Support for Participants and Study Staff

Providing adequate training and support to participants on how to use the eDiary app effectively reduces technical barriers and increases participant satisfaction [15]. During the initial visit, the participants should be trained by the trial staff on how to use the eDiary app, encouraged to ask any questions, and educated on the importance of timely reporting of ARs. The training may include hands-on tutorials, frequently asked questions, and on-site guidance during the first visit.

It is equally important to train trial staff on using the web-based eDiary database, setting up participant accounts, monitoring daily participant compliance, and troubleshooting the eDiary app when needed. Training backup personnel for when the primary trial staff are not available will support uninterrupted data collection. Having accurate training manuals and user guides before data collection is very important. Lack of clear protocols, measures, or proper training of trial staff may result in biased or inconsistent data in the clinical trials [16].

### Adherence and Compliance

Setting up regular reminders and prompts for participants to complete their eDiary entries aids compliance efforts. These reminders can be sent via email, text messages, or push notifications within the eDiary app to encourage timely and consistent reporting throughout the reporting window [17]. Email notifications can be sent to the participants who did not complete their eDiaries within the reporting time window. In addition, alerts for participants who enter symptoms of greater severity may include instructions to contact the trial staff as soon as possible for further evaluation.

Automated notifications may be sent to the trial staff with a list of participants who did not complete their eDiaries within the reporting time, allowing the site staff to follow up immediately. When a participant reports a symptom with severity above a predetermined threshold as per the protocol, or if a symptom gets worse, real-time automated alerts and notifications can be sent to the trial staff to facilitate further evaluation in a timely manner.

### Data Quality Monitoring

Implementing data quality monitoring procedures to ensure the accuracy and completeness of AR reporting plays an important role. This may involve regular data checks (in the majority of the vaccine trials, daily monitoring of compliance is crucial), outlier detection, and validation of data to ensure that data adhere to ALCOA standards.

### Daily Questionnaire

Participants should be provided a clear and comprehensive list of solicited ARs of interest for the specific vaccine or trial. This list should include common and expected ARs, along with their definitions and grading criteria.

### Reporting Window

Setting a defined reporting time window in eDiaries is critical for collecting high-quality safety data in clinical trials. It ensures that participants are reporting ARs within a consistent timeframe and allows for more meaningful comparisons between participants. A defined reporting window is the length of time in which participants may complete their daily eDiary questionnaire (eg, 6 PM to 11:59 PM). Restricting the functionality of the eDiary once the reporting window ends helps avoid inaccurate readings due to recall bias. A recall bias occurs when a participant does not recall details accurately, such as a temperature reading or the entire occurrence of an event, leading to incomplete or inaccurate data entry. Appropriate reporting time windows should be selected based on the trial protocol and objective to balance the need for accurate data collection and minimize both missing data and the burden on participants [4,18].

### Date and Time Stamps

One of the biggest advantages of eDiaries is date and time stamping of diary entries without the need to forward or backward fill. Date and time stamping ensures accurate data collection and minimizes the occurrence of inaccurate entries seen with paper diaries [4,19]. For example, if the participant starts the day-2 eDiary on day 2 at 11:45 PM but finishes on day 3, recording both the start and end date and time stamps helps with meaningful data interpretation and analysis. This is important because, if the participant enters the data corresponding to day 2 on day 3, trial staff may perceive it to be less accurate and less reliable due to recall bias.

### Data Confidentiality and Security

Robust data security measures should be implemented to protect participant confidentiality and privacy. These measures include secure data storage, encryption, and access controls to prevent unauthorized access or data breaches.

### Patient Engagement

Providing regular feedback to participants regarding their AR reporting, acknowledging their contributions, and promptly addressing any concerns or questions fosters engagement and encourages continued participation.

Evaluating eDiary processes regularly and collecting feedback from participants are important to identify areas for improvement and make modifications as needed.

### Handling Missing Data

eDiaries can minimize missing data, with real-time monitoring of participant compliance, where simultaneous observation of data is possible as it is reported, enabling timely interventions by study personnel. However, sometimes it may be inevitable that the collected data have missing values for solicited local and systemic ARs during the duration of the collection period. While designing a user-friendly eDiary interface and setting reminder or alert systems are some early measures to minimize missing data, existing missing data may be handled by identifying patterns, such as whether the missing data are random or clustered around a specific symptom, population, or day. Performing sensitivity analyses to assess the potential impact of missing data on the results helps to ensure the integrity of the findings.

In addition to the above-listed measures, early engagement with regulatory agencies regarding the use of these tools to solicit ARs in vaccine clinical trials is crucial to ensure that eDiaries are used as intended and align with regulatory expectations.

## Hypothetical Scenarios

### Scenario Framework

Use of an eDiary without adequate training and quality control measures can compromise data integrity and jeopardize the trial’s purpose. Below, we present 2 hypothetical scenarios to demonstrate how the strategies listed above may help avoid some of the common challenges associated with using eDiaries for data collection. The selection of hypothetical scenarios is based on common issues that have been identified in previous clinical studies. The scenarios are designed to be relevant, realistic, and provide insights into potential challenges and opportunities associated with eDiary use in vaccine clinical trials.

### Hypothetical Scenario 1: Reporting Window

#### Scenario Introduction

A pharmaceutical company launched a phase 3 clinical trial assessing the safety and immunogenicity of a vaccine against an infectious disease. The company planned to use eDiaries to collect safety data for 14 days postvaccination.

#### Overlooked Crucial Steps

However, the company overlooked a crucial step: the reporting window was extensive. The eDiary reporting window was scheduled to open at 6 AM on the respective trial day or the day from vaccination. For example, the day-2 eDiary opened at 6 AM on trial day 2, the day 3 eDiary opened at 6 AM on trial day 3, and so on. If the participant did not fill in the eDiary by 11:59 PM of the trial day, the missed eDiary would be available for the next 3 days under the “Missed eDiaries” tab of the eDiary app. Study staff were trained to monitor participant compliance frequently, but to contact the participant regarding any missed entries only after the 3-day extended reporting window was closed.

#### Consequences

Participants who did not complete their day-2 eDiary were reminded by the trial staff to fill in the missed eDiary after the 3-day extended reporting window (day-3 to day-5) was closed, which is on day 6 after vaccination. However, some participants struggled to remember their symptoms from day 2, especially temperature readings and local reactions, resulting in inaccurate and missing data.

#### Lessons Learned

Large reporting windows may contribute to recall bias, leading to inaccuracies and missing data. This issue could be avoided by extending the window slightly beyond the 24-hour interval. For instance, trial day 2 eDiary could be made available from 6 AM on day 2 until 11 AM on day 3. Furthermore, trial staff should be trained to monitor participant compliance daily and contact noncompliant participants immediately to avoid any recall bias. To streamline the process, an alert system involving emails and SMS text messages to trial staff with the list of participants who are noncompliant may help determine who needs to be contacted immediately.

Having a large reporting window might also hinder the advantage of eDiary usage for real-time monitoring of participant compliance. It is ideal to collect the symptoms within a 24-hour interval. Despite efforts to capture everything, the possibility of missing data exists. In such cases, it would be beneficial to perform a sensitivity analysis to investigate the impact of AR reporting within the 24-hour interval versus outside the 24-hour interval on the overall safety outcome.

### Hypothetical Scenario 2: Training Study Staff and Participants

#### Scenario Introduction

A pharmaceutical company launched a phase 1/2 clinical trial assessing the safety and immunogenicity of a vaccine in children 5 to 11 years old. The company planned to use eDiaries to collect safety data for 7 days postvaccination.

#### Overlooked Crucial Steps

Two things were overlooked in this trial. First, the trial staff were not properly trained to regularly monitor participant compliance or instructed to contact participants for any noncompliance. Second, when a grade 3 or more AR was reported, the eDiary produced a pop-up message to contact trial staff. However, the participants were not appropriately trained to understand the severity grades of solicited ARs, and the importance of their timely reporting to the trial staff was not communicated properly.

#### Consequences

Due to poor training, the trial staff did not monitor compliance frequently or ask participants to complete their eDiary reports on time, which led to inaccurate, incomplete, and missing data. Moreover, complete responsibility was put on the participants to report severe ARs and contact trial staff for further clinical evaluation, without proper knowledge of the severity grading criteria and the importance of timely reporting. As a result, inconsistencies in grading ARs between the participant and the investigator were identified.

#### Lessons Learned

Training the trial staff and participants (or the legally authorized representatives in pediatric trials) is very crucial for capturing meaningful and reliable data [16].

## Discussion

The efficiency and accuracy of clinical trials hinge on robust data collection processes. Compliance and accuracy are important features to generate meaningful data when diaries are used to collect PROs. One of the problems with paper diaries is nonadherence to the protocol, as shown in a study by Stone et al [20], where participant-reported compliance by paper diary was more than 90%; however, actual compliance was only 11%, as captured by eDiary, when reporting pain. Paper diaries have high recall bias, lower adherence, and, hence, limited reliability [21]. ePROs have revolutionized this aspect, offering streamlined data collection and real-time monitoring capabilities. eDiary reporting offers several advantages, including date and time stamping of diary entries, data completeness, accuracy, instant access to data, and user acceptance [22]. Better participant compliance with eDiary reporting has been reported in multiple studies [22-24]. Some of the measures that helped with high eDiary compliance include the ability to set up alarms or reminders within eDiary apps, shorter diaries due to skip logic (fewer questions), and participant training manuals [15].

Asking participants their reasons for noncompliance and reporting them may help improve compliance and help with the development of strategies to overcome missing data. Reported recommendations that might motivate participants to complete the daily eDiary questionnaire include design elements such as having a thank-you screen at the end of the daily questionnaire, a progress tracker as participants complete the questionnaire, information on the first screen comprising the summary of daily questions and estimated time to complete, and in-app alerts to inform if user compliance is below expectations [25]. Length of the trial may impact participant compliance, which may decrease significantly from the first to second week of eDiary reporting (83.4% to 76.7%; *P*<.001), as seen in the study by Jiang et al [24]. Gamification and in-app rewards can enhance participant engagement with eDiaries by incorporating points and prizes, making the data collection more engaging and motivating [15,26]. The reporting window time may hinder compliance based on the participant’s personal schedule; evening compliance was reported to be best compared to afternoon or morning reporting times [24]. Hence, it is important to define the reporting windows and trial length carefully to reduce disruption to the participant’s daily life. In the same trial, other reasons that contributed to poor diary compliance were identified, including lack of awareness that reported information would be used to guide asthma care and directly influence overall health care or unreasonable frequency of diary entries per day [12].

Despite the promising features of the eDiary app to collect PROs data, they do not provide a one-size-fits-all solution. Participants may face problems due to age-related factors, socioeconomic status, or their digital literacy when using eDiaries to report data. Hence, it is very important to tailor strategies based on trial objectives and study population, such as increasing the font size within the app and providing additional training, when needed, if most of the trial population is older.

## Conclusions

In this paper, we present our reflections on strategies that would mitigate commonly encountered challenges when using eDiaries for reliable and accurate safety data collection. By demonstrating a commitment to safety throughout the development and approval processes, researchers and regulatory agencies can contribute to vaccine acceptance and improve public health outcomes. It is important to note that the FDA does not regulate the methods or techniques used in study activities; rather, it ensures that studies are conducted per regulation and guidance. In other words, recommendations are made to ensure data completeness during clinical trials, but not to dictate which technique to be used. Across FDA guidance, the importance of data integrity, including completeness, is reiterated, and sponsors are encouraged to leverage the available state-of-the-art methods and techniques to achieve this goal.

## Abbreviations

ALCOA: Attributable, Legible, Contemporaneous, Original, Accurate
ALCOA+: Attributable, Legible, Contemporaneous, Original, Accurate, Complete, Consistent, Enduring, and Available
AR: adverse reaction
eDiary: electronic Diary
ePRO: electronic patient-reported outcome
FDA: Food and Drug Administration
PRO: patient-reported outcome
